# Role of Betaglycan in TGF-β Signaling and Wound Healing in Human Endometriotic Epithelial Cells and in Endometriosis

**DOI:** 10.3390/biology11040513

**Published:** 2022-03-26

**Authors:** Agnes N. Mwaura, Muhammad A. Riaz, Jane B. Maoga, Ezekiel Mecha, Charles O. A. Omwandho, Georgios Scheiner-Bobis, Ivo Meinhold-Heerlein, Lutz Konrad

**Affiliations:** 1 Center of Gynecology and Obstetrics, Faculty of Medicine, Justus-Liebig-University, Feulgenstr. 10-12, D-35392 Giessen, Germany; agnes.mwaura-njoki@med.uni-giessen.de (A.N.M.); muhammad.a.riaz@gyn.med.uni-giessen.de (M.A.R.); jane.maoga@med.uni-giessen.de (J.B.M.); ivo.meinhold-heerlein@gyn.med.uni-giessen.de (I.M.-H.); 2 Department of Biochemistry, University of Nairobi, Nairobi 00100, Kenya; emecha@uonbi.ac.ke; 3 Reproductive Biochemistry, Kirinyaga University, Kerugoya 10300, Kenya; omwandho@kyu.ac.ke; 4 Institute for Veterinary Physiology and Biochemistry, School of Veterinary Medicine, Justus-Liebig-University, D-35392 Giessen, Germany; georgios.scheiner-bobis@vetmed.uni-giessen.de

**Keywords:** TGF-βs, betaglycan, SMAD2/3, endometriosis, wound healing

## Abstract

**Simple Summary:**

Endometriosis is a benign female disorder presumably caused by dislocation of endometrial tissue during menstruation most often into the pelvis and is mainly resulting in pain and infertility. Up to date the pathogenesis of endometriosis is still unclear and a non-invasive biomarker not available. One of the cytokines suggested to be involved in the pathogenesis are the transforming growth factor-betas 1-3, which signal via two high-affinity receptors and in the case of transforming growth factor-beta2 also via the co-receptor betaglycan. In this study, we have analyzed the involvement of betaglycan into transforming growth factor-beta signaling and the contribution to biological functions such as wound healing with an in vitro model. We demonstrated an interesting interaction of betaglycan with the transforming growth factor-betas 1-3 and vice versa. Remarkably, the co-receptor betaglycan in its soluble form reduced wound healing as well as secretion of transforming growth factor-betas. Although we found some hints that endocervical mucus levels are different between healthy subjects and cases with endometriosis it was not sufficient for a reliable non-invasive diagnosis of the disease. Taken together, our findings suggest a novel role for betaglycan in the pathogenesis of endometriosis.

**Abstract:**

Endometriosis is characterized by the presence of ectopic endometrium most often in the pelvis. The transforming growth factor-beta (TGF-β) superfamily is also involved in the pathogenesis; however, betaglycan (BG, syn. TGF-β type III receptor) as an important co-receptor was not studied. We analyzed mainly BG ectodomain shedding because released soluble BG (sBG) often antagonizes TGF-β signaling. Furthermore, we studied the role of TGF-βs and BG in wound healing and evaluated the suitability of BG measurements in serum and endocervical mucus for non-invasive diagnosis of endometriosis. Evaluation of the BG shedding and signaling pathways involved as well as wound healing was performed with enzyme-linked immune assays (ELISAs), reverse transcription-quantitative polymerase chain reaction (RT-qPCR), small interfering RNA (siRNA) knockdown, and scratch assays with human endometriotic epithelial cells. TGF-β1/2 stimulation resulted in a significant dose-dependent reduction in BG shedding in endometriotic cells, which was TGF-β/activin receptor-like kinase-5 (ALK-5)/mother against decapentaplegic homolog3 (SMAD3)- but not SMAD2-dependent. Inhibition of matrix metalloproteinases (MMPs) using the pan-MMP inhibitor GM6001 and tissue inhibitor of MMPs (TIMP3) equally attenuated BG shedding, signifying the involvement of MMPs in shedding. Likewise, recombinant BG moderately reduced the secretion of TGF-β1/2 and wound healing of endometriotic cells. TGF-β1 significantly enhanced the secretion of MMP2 and MMP3 and moderately promoted wound healing. In order to evaluate the role of BG in endometriosis, serum (n = 238) and mucus samples (n = 182) were analyzed. Intriguingly, a significant reduction in the levels of sBG in endocervical mucus but not in the serum of endometriosis patients compared to controls was observed. Collectively, these observations support a novel role for BG in the pathophysiology of endometriosis.

## 1. Introduction

Approximately 0.7–8.6% of reproductive-age women suffer from endometriosis, a chronic estrogen-dependent gynecological condition, typically associated with chronic pelvic pain, dyspareunia, dysmenorrhea, dyschezia, and infertility [1,2,3]. The condition is characterized by the occurrence of endometrial tissue outside the uterine cavity, primarily in the ovaries, pelvic peritoneum, bladder, bowel, and retro-vaginal septum [2,3]. Although considered benign, endometriosis exhibits features similar to that of malignant tumors including cell growth, cell migration and invasion, neo-vascularization, and reduced apoptosis [2,3,4,5]. Without a reliable non-invasive marker, to date, only laparoscopy followed by histological evaluation results in a definitive diagnosis [1,2,3]. However, recently the study of cervical mucus has shown some potential [6,7].

The involvement of transforming growth factor betas (TGF-βs) in the pathogenesis of endometriosis was recently reviewed [4,8,9]. The TGF-β superfamily comprises TGF-βs, activins, inhibins, bone morphogenetic proteins (BMPs), along with growth and differentiation factors (GDFs) [10]. They are fundamental to normal cellular functions such as cell proliferation, survival, differentiation, matrix production, motility, angiogenesis, apoptosis, and immune modulation [4,8,9,11]. All members of the TGF-β superfamily signal via pairs of serine/threonine kinase receptors; in the case of the TGF-βs, type I and II TGF-β receptors (TβRI and TβRII), assemble into heteromeric complexes on the cell surface [10]. TGF-β ligands bind to their respective cell surface receptors followed by phosphorylation and activation of specific downstream targets [12,13]. In the canonical TGF-β signaling pathway, the TGF-β ligands activate members of the SMAD transcription factor family, especially SMAD2/3, and after binding to the common SMAD4 followed by translocation to the nucleus, target genes are transcribed [12].

Betaglycan (BG, syn. TβRIII) is a ubiquitously expressed transmembrane co-receptor for some TGF-β superfamily ligands [8,14,15,16]. It is an 851-amino acid heparan sulfate proteoglycan with a large extracellular domain, a single-pass hydrophobic transmembrane domain, and a short cytoplasmic domain lacking kinase activity [14,16]. BG functions to establish the potency of its ligands, chiefly TGF-β2 and inhibin A, on the target cells [16] and has other additional ligand-dependent and -independent roles in the regulation of reproduction and tumor suppression [17,18,19,20]. BG null embryos present with cardiovascular and hepatic defects are not viable and die between embryonic day 13.5 and birth [21,22]. In the uterus, BG is expressed in endometrial glands and endothelial cells [23]. Dysregulated TGF-β expression is involved in the pathogenesis of endometriosis and other diseases [4,8,24].

Membrane-bound BG undergoes proteolytic ectodomain cleavage, a process termed shedding, releasing a soluble domain (sBG) that can be detected in the extracellular matrix and body fluids such as milk, serum, and plasma [20,25,26,27]. Notably, restoring or increasing BG expression in numerous cancer models, via administration of sBG, decreases cancer cell motility and invasiveness in vitro [19,28] and invasiveness, angiogenesis, and metastasis in vivo [19,29,30,31].

Although BG has been demonstrated to participate in multiple diseases [17,19,20,32,33] including endometrial cancer [23,33,34], its role in endometriosis is unknown. Thus, we investigated the involvement of BG in endometriosis with endometriotic 12Z cells in vitro, explored the modulation of BG shedding by TGF-β1/-β2, TIMPs and MMPs, and the influence of TGF-βs and BG on wound healing. The suitability of serum/endocervical mucus sBG levels as a non-invasive diagnostic biomarker for endometriosis was also tested.

## 2. Materials and Methods

### 2.1. Cell Lines and Cell Culture

The immortalized and well-characterized 12Z cell line [35] was kindly provided by Prof. Anna Starzinski-Powitz (Department of Biology, University of Frankfurt, Frankfurt, Germany) and is frequently used by many researchers to study endometriotic epithelial cells [36,37]. 12Z cells are regularly tested for epithelial markers such as mucin-1, keratins etc., functional characteristics such as hormone responsiveness, and contamination with mycoplasmas as published [38]. Cells were maintained in medium as follows: 12Z cells in DMEM 4.5 g/L glucose supplemented with 10% FCS, 2 mM glutamine, and 1% penicillin/streptomycin (pen-strep) and cultured in a humidified incubator at 37 °C and 5% CO_2_. The medium was routinely renewed every 3–4 days. Cells were washed once with 1 × PBS without Ca^2+^ and Mg^2+^ before detachment with 0.25% accutase and passaged at approximately 80% confluence. Cells seeded in culture well plates were serum-starved for 24 h before starting the experiments. All cell culture reagents were obtained from Invitrogen/ThermoFisher Scientific (Karlsruhe, Germany).

### 2.2. Recombinant Proteins, Inhibitors, and ELISA Kits

The following materials were used: recombinant human TGF-β1 and TGF-β2 (Promokine, Heidelberg, Germany); recombinant human betaglycan, recombinant human TIMP3 (both from R&D Systems, Wiesbaden, Germany); recombinant human TIMP1 and TIMP2 (both from SinoBiological, Eschborn, Germany) Halt™ Protease Inhibitor Cocktail (100×) (ThermoFisher Scientific, Carlsbad, CA, USA); Cell lysis buffer (Cell signaling technology, Frankfurt, Germany); LY364947, GM6001 (both from Sigma Aldrich, St. Louis, Missouri, USA); Sample activation Kit 1 (R&D Systems); human TGF-beta RIII DuoSet ELISA kit (DY242, range 156–10,000 pg/mL); human TGF-beta 1 DuoSet ELISA kit (DY240, range 31.3–2000 pg/mL); human TGF-beta 2 DuoSet ELISA kit (DY302, range 31.3–2000 pg/mL); Human MMP-2 DuoSet ELISA kit (DY902, range 0.6–20 ng/mL); Human Total MMP-3 DuoSet ELISA kit (DY513, range 31.3–2000 pg/mL). All ELISA kits were purchased from R&D Systems.

### 2.3. Collection of Supernatants for ELISAs

Cells (2 × 10^5^ cells) cultured in 6-well plates for 24 h and serum-starved (1% FCS) for 24 h were treated in duplicate with TGF-β1 (1–15 ng/mL), TGF-β2 (1–15 ng/mL), betaglycan (10–100 ng/mL), TIMP1 and TIMP2 (both 10–200 ng/mL), TIMP3 (2.5–10 nM) or GM6001 (10 µM). For inhibition studies, cells were pre-treated with the inhibitor for 2 h (10 µM, LY364947 diluted in 0.01% DMSO) or 72 h (SMAD2/SMAD3 siRNA gene knockdown) prior to stimulation with TGF-β1 or TGF-β2 (each 10 ng/mL). Negative controls consisted of untreated samples without the recombinant proteins or containing the vehicle (0.01% DMSO) or control siRNA. Cell culture supernatants were collected in Eppendorf tubes with 1 × Halt™ protease inhibitor cocktail and stored at −20 °C until use. For normalization of ELISA concentrations, cells were washed once with 1 × PBS and detached with 500 µL/well accutase for 3 min at 37 °C before suspending in the medium. The single cell suspension (10 µL) was then stained with Trypan Blue (10 µL) and the number of viable cells assessed with the TC10™ automated cell counter system (Bio-Rad, Dusseldorf, Germany). For preparation of cell lysates, supernatants were collected as described before, and cells were washed with ice-cold PBS before lysis with lysis buffer containing 1 × Halt™ protease inhibitor cocktail. Then, lysates were collected with a cell scraper and sonicated for 5 sec before centrifugation at 13,000× *g* for 15 min at 4 °C. Protein concentrations were determined with the Bicinchoninic acid (BCA) protein assay kit (Pierce, ThermoFisher Scientific, Carlsbad, CA, USA) following the manufacturer’s instructions.

### 2.4. TGF-Beta RIII DuoSet ELISA

Cells were treated and supernatants collected as described and then analyzed with the human TGF-beta RIII DuoSet ELISA kit according to the manufacturer’s instructions. Briefly, the ELISA plate (96 wells, ThermoFisher Scientific, Carlsbad, CA, USA) was coated overnight with 4 μg/mL capture antibody diluted in 1 × PBS pH 7.2 at 4 °C. Plates were blocked by adding 300 μL of reagent diluent to each well and incubated at room temperature (RT) for a minimum of 1 h. Afterwards, 100 μL supernatant and TGF-beta RIII standards in reagent diluent were added and incubated for 2 h at RT. After an additional 2h incubation step with 400 ng/mL detection antibody, streptavidin-HRP solution (1:200) was added to the wells for 20 min. Then, substrate solution (100 µL) was added for 20 min and the reaction stopped with 50 µL 2N H_2_SO_4_. Values were obtained with an infinite^®^ 200 microplate reader (TECAN, Männedorf, Switzerland) set at 450/540 nm. Soluble BG concentrations were standardized against the number of viable cells or the total protein concentrations of the corresponding lysates.

### 2.5. SiRNA Transfection

Silencer select siRNA constructs for SMAD2/3 and control as well as transfection reagents were purchased from ThermoFisher Scientific (Carlsbad, CA, USA). Epithelial 12Z cells (1 × 10^5^ cells/mL) were grown to 60% confluence in 12-well plates and transfected with transfection reagent alone, silencer™ select negative control No. 2 siRNA (Cat. No., 4390847), SMAD2 siRNA (siRNA ID #: 107873) or SMAD3 siRNA (siRNA ID #: s535081) using Lipofectamine™ RNAiMAX transfection reagent in medium without antibiotics according to the manufacturer’s guidelines. Briefly, siRNA and transfection reagent were diluted 1:3, siRNA: Lipofectamine each in 150 µL Opti-MEM medium (ThermoFisher Scientific, Carlsbad, CA, USA). Equal parts of diluted siRNA and diluted Lipofectamine were then mixed and incubated for 5 min at room temperature. The mixture (300 µL) was then added to the cells (700 µL antibiotic-free medium) to achieve a final siRNA concentration of 100 nM (SMAD2 siRNA) or 150 nM (SMAD3 siRNA). Then medium was changed for complete growth medium after 24 h and cells incubated for 48 h. Efficiency of the siRNA gene knockdown was determined after every transfection procedure by RT-qPCR.

### 2.6. RNA Isolation and Real Time-qPCR

Total RNA was extracted from transfected 12Z cells using the RNAeasy Kit (Qiagen, Hilden, Germany) in accordance to the manufacturer’s protocol. RNA quantity and quality were determined using a P300 NanoPhotometer^®^ (Implen, Munich, Germany). A First-Strand cDNA Synthesis kit (Thermo/Scientific, Carlsbad, CA, USA) was then used to reverse-transcribe the RNA (1 µg) following the supplier’s instructions. PCR primers (Thermo/Scientific, Carlsbad, CA, USA Table 1) were designed using the NCBI Primer-Blast algorithm available at http://www.ncbi.nlm.nih.gov/tools/primer-blast (accessed on 01 March 2021). RT-qPCR was conducted with 0.5 µg cDNA in a 20 µL PCR reaction using iTaq™ Universal SYBR^®^ Green Supermix on a MiniOpticon™ Real-Time PCR System (Bio-Rad, Dusseldorf, Germany). The cycling program was initiated by a first denaturation at 95 °C for 5 min, followed by 35 cycles of denaturation at 95 °C for 30 seconds (s), annealing at 59 °C for 20 s, and a final elongation step at 72 °C for 30 s except for the final extension that lasted 10 min. GAPDH was used for normalization.

### 2.7. TGF-β1/-β2 and MMP-2/3 ELISAs

Levels of secreted TGF-β1, TGF-β2, MMP2, and MMP3 in supernatants were determined by ELISAs. For the TGF-β1 and TGF-β2 ELISAs, samples were activated with 1N HCl and then neutralized with 1.2N NaOH/0.5M HEPES. ELISAs were performed according to the supplier’s guidelines.

### 2.8. Wound Healing Assay

The scratch assay according to Liang et al. [39] was used with some modifications. Briefly, 12Z cells were cultured in six-well plates and the confluent cell monolayer disrupted by scratching with a sterile 200 µL pipette tip. Culture medium was immediately removed and after washing with 1 × PBS, cells were grown in serum-free medium with or without 10 ng/mL TGF-β1, -β2 or 25 ng/mL betaglycan. Migration of cells into the cell-free areas was monitored at 0, 24, and 48 h by capturing images under a Leica DM IL microscope fitted with a Canon EOS 450D camera. Four fields were analyzed per well using ImageJ software version 1.53 h accessed on 01 March 2021 (http://rsbweb.nih.gov/ij/).

### 2.9. Patients

The current study was approved by the Ethics Committee of the Medical Faculty of the Justus-Liebig-University, Giessen, Germany (registry number 95/09). Informed written consent was obtained from all participants prior to sample collection in accordance with the approved protocol. The study group for serum sBG consisted of 166 women with and 72 women without endometriosis while that of endocervical mucus sBG consisted of 100 women with and 82 women without endometriosis (Table 2). Patients on hormone therapy consisted of women taking dienogest- or dienogest plus ethinylestradiol- or progestin-based medications. Diagnosis of endometriosis was performed by laparoscopic surgery followed by histological confirmation.

### 2.10. Sample Collection and Analysis

Two to three milliliters of anticoagulant-free venous blood was obtained from each subject (Table 2 and Table 3) during clinical examination. Samples were centrifuged at 3000× *g* for 15 min at 4 °C and stored immediately as aliquots at −80 °C until use. Endocervical mucus samples from patients (Table 2 and Table 3) were obtained in the afternoon (2–4 pm) with a cotton swab and immediately placed in ice-cold 1 × PBS containing 1 × protease inhibitor cocktail (P1860, Sigma, Taufkirchen, Germany). Samples were then centrifuged at 3000× *g* for 15 min at 4 °C and supernatants collected, weighed, and stored as aliquots at −80 °C until further analysis. In all cases, the use of contraception and menstrual cycle phases were monitored by anamnesis and a questionnaire [40]. Levels of sBG in serum and endocervical mucus were detected using the human TGF-beta RIII DuoSet ELISA kit according to the manufacturer’s guidelines.

A guide through the experimental setup can be found in Figure S1 (Supplementary Material).

### 2.11. Statistical Analyses

All statistical analyses were performed with GraphPad Prism software (Version 8.0, GraphPad Inc. La Jolla, CV, USA). Each in vitro experiment was performed at least three times in duplicate. Statistical comparisons of the means between two groups were done using t-tests and among multiple groups by one-way analysis of variance (ANOVA) followed by Dunnett’s post hoc tests. Analyses of serum and endocervical sBG levels were performed with the Mann–Whitney U-test (for two group comparisons) or the Kruskal–Wallis test (for more than two groups). A receiver operating characteristics (ROC) curve and area under the curve (AUC) with a 95% confidence interval was calculated to define the optimal cut-off for endocervical mucus sBG levels. Then, a 2 × 2 contingency table was used to calculate sensitivity, specificity, and the positive likelihood ratio. Differences were considered statistically significant at *p* ≤ 0.05. All data are reported as means ± SEMs (SEM = standard error of the mean).

## 3. Results

### 3.1. Modulation of BG Shedding in Endometriotic Epithelial Cells by TGF-βs

**Figure 1:**
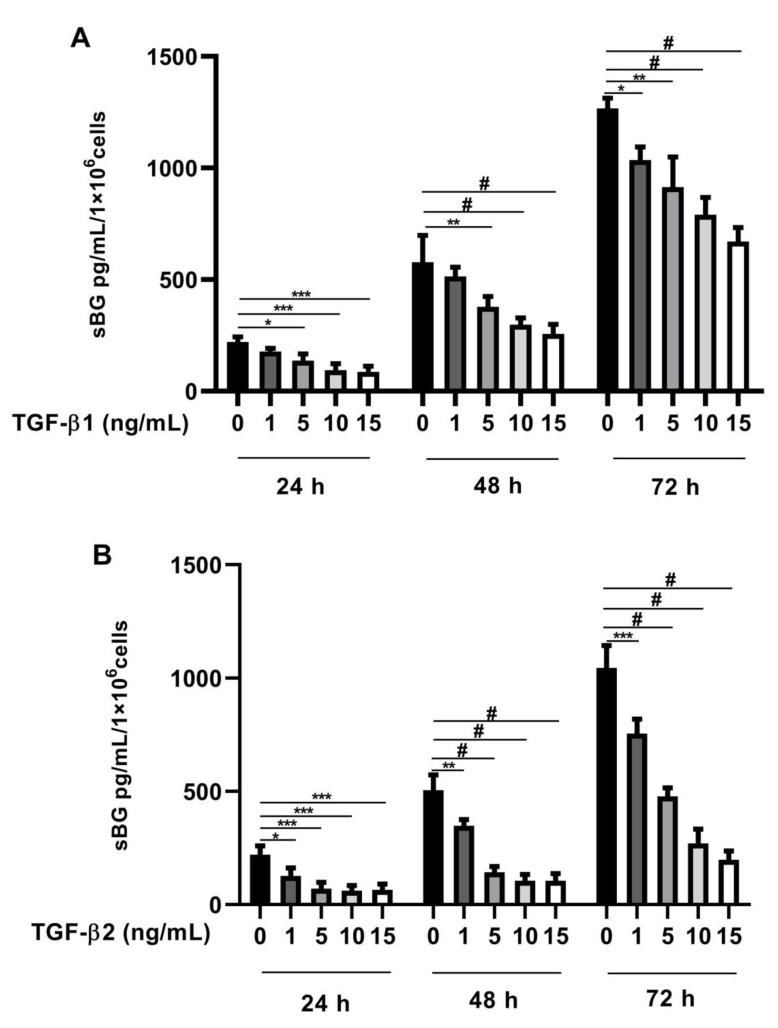
Dose-dependent effects of TGF-β1 and -β2 on BG shedding. Epithelial 12Z cells were stimulated with increasing concentrations of TGF-β1 (**A**) or TGF-β2 (**B**) (1–15 ng/mL) for 24 h, 48 h, and 72 h; supernatants were analyzed by sBG ELISA. Both TGF-β1 and -β2 treatment reduced betaglycan shedding in a dose-dependent and significant manner. Each bar represents the mean ± SEM of three independent experiments performed in duplicate. * *p* ≤ 0.05; ** *p* < 0.01; *** *p* < 0.001; ^#^ *p* < 0.0001; sBG, soluble betaglycan.

Recently, we found that BG is a vital modulator of TGF-β2 signaling in Sertoli cells [41]. In the present study, we investigated whether TGF-βs regulate BG shedding in human endometriotic 12Z cells. Firstly, we evaluated TGF-β concentration dependency in BG shedding and found a significant dose-dependent decrease in BG shedding after stimulation with TGF-β1 (Figure 1A) and TGF-β2 (Figure 1B) compared to controls after 24, 48, and 72 h. Remarkably, a stronger reduction in BG shedding was observed with TGF-β2 compared to TGF-β1 (Figure 1A,B). Because the strongest effects were observed with 10 ng/mL TGF-β1 and 10 ng/mL TGF-β2, the following experiments were conducted with these concentrations.

**Figure 2:**
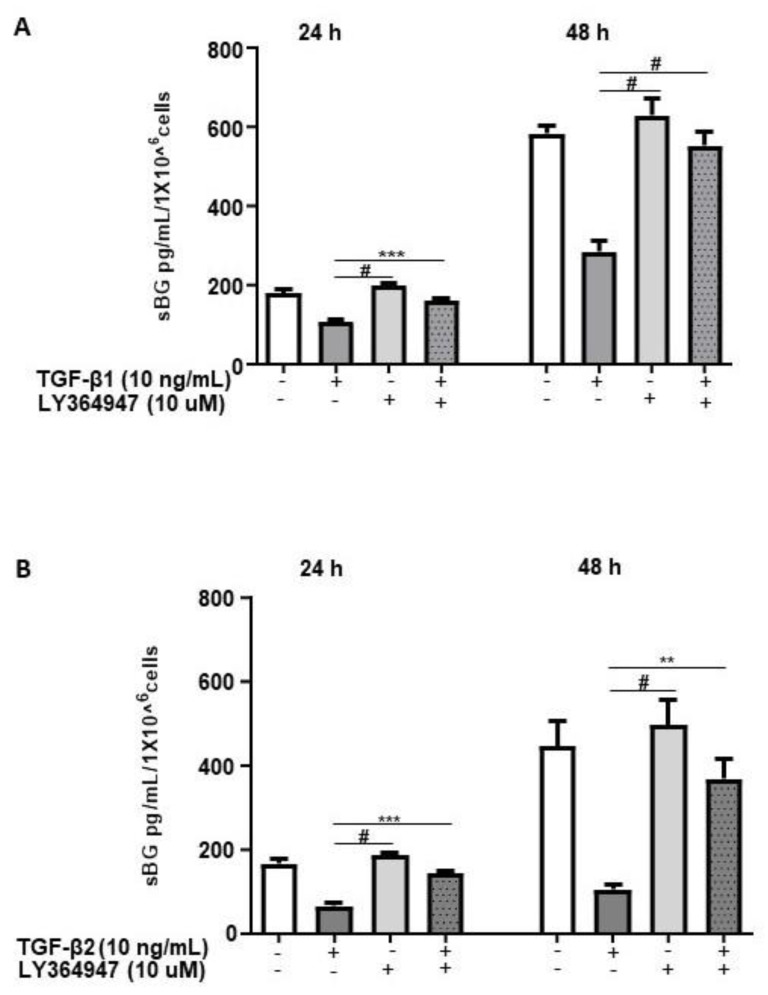
TGF-β1/2-mediated reduction in BG shedding is TGF-β type 1 receptor (ALK-5)-dependent. Epithelial 12Z cells were pre-incubated in the absence or presence of ALK-5 inhibitor, LY364947 (10 µM) for 2 h prior to stimulation with TGF-β1 (10 ng/mL) (**A**) or TGF-β2 (10 ng/mL) (**B**) for 24 and 48 h; supernatants were analyzed by sBG ELISA. LY364947 significantly abrogated the reduction in BG shedding induced by TGF-β1 and -β2. Each bar represents the mean ± SEM of three independent experiments performed in duplicate. ** *p* < 0.01; *** *p* < 0.001; ^#^ *p* < 0.0001; sBG, soluble betaglycan.

TGF-βs exert their biological functions mainly via the canonical SMAD-signaling pathway and to a lesser extent via non-canonical pathways [12]. Thus, we investigated the pathways involved in TGF-β-mediated effects on BG shedding. 12Z cells pretreated with LY364947, a selective inhibitor of TGF-β type I receptor (ALK-5), demonstrated that LY364947 significantly attenuated the TGF-β1 and TGF-β2-induced reduction in BG shedding (Figure 2A,B).

**Figure 3:**
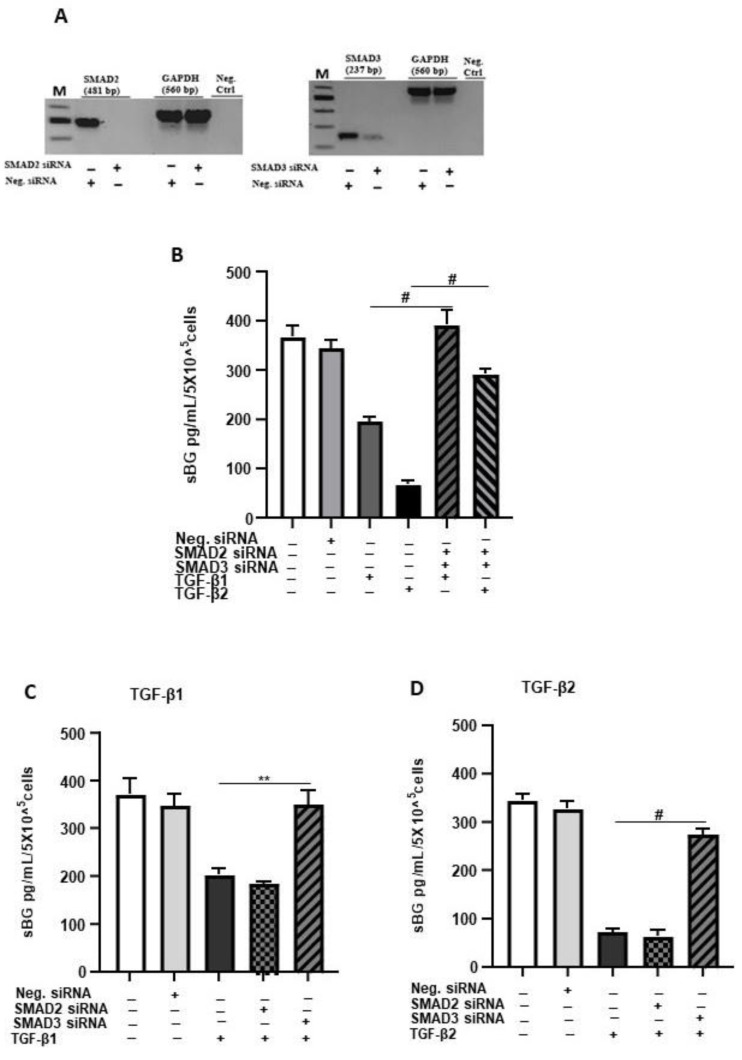
TGF-β1/2-mediated reduction in BG shedding is SMAD3- but not SMAD2-dependent. Epithelial 12Z cells were plated in 6-well plates and transfected with SMAD2 siRNA, SMAD3 siRNA or negative control siRNA and a control RT-qPCR of the gene silencing efficiency for each siRNA performed to ascertain silencing of SMAD2 and SMAD3 (**A**). SMAD2/3 double-silenced (**B**) and single-silenced (**C**,**D**) epithelial 12Z cells were stimulated with TGF-β1 or TGF-β2 (10 ng/mL) for 48 h, and supernatants were analyzed by sBG ELISA. SMAD2/SMAD3 double gene knockdown significantly abrogated the TGF-β1 and TGF-β2-dependent reduction in BG shedding (**B**). Analysis of individual knockdowns revealed that silencing of SMAD3 gene and not of SMAD2 gene abolished the TGF-β1- and TGF-β2-mediated reduction in BG shedding (**C**,**D**). Each bar represents the mean ± SEM of three independent experiments performed in duplicate. ** *p* < 0.01; ^#^ *p* < 0.0001; M, DNA ladder; Neg. siRNA, control siRNA; Neg. Ctrl, negative control; bp, base pairs; sBG, soluble betaglycan.

Next, we explored the involvement of SMAD2/3 signaling in TGF-β-mediated reduction in BG shedding using gene silencing (Figure 3). The SMAD2/SMAD3 double gene knockdown significantly ameliorated the TGF-β-dependent reduction in BG shedding (Figure 3B). The individual knockdowns revealed for SMAD3 ~100% and ~85% inhibition of the TGF-β1 and TGF-β2 effects, respectively, on BG shedding. In contrast, silencing of the SMAD2 gene had no effects (Figure 3C,D). Thus, the canonical TGF-β pathway involving TGF-β/ALK-5/SMAD3 signaling is required in TGF-β-mediated reduction in BG shedding in 12Z cells.

### 3.2. Effects of Recombinant BG on TGF-β1 and TGF-β2 Secretion

**Figure 4:**
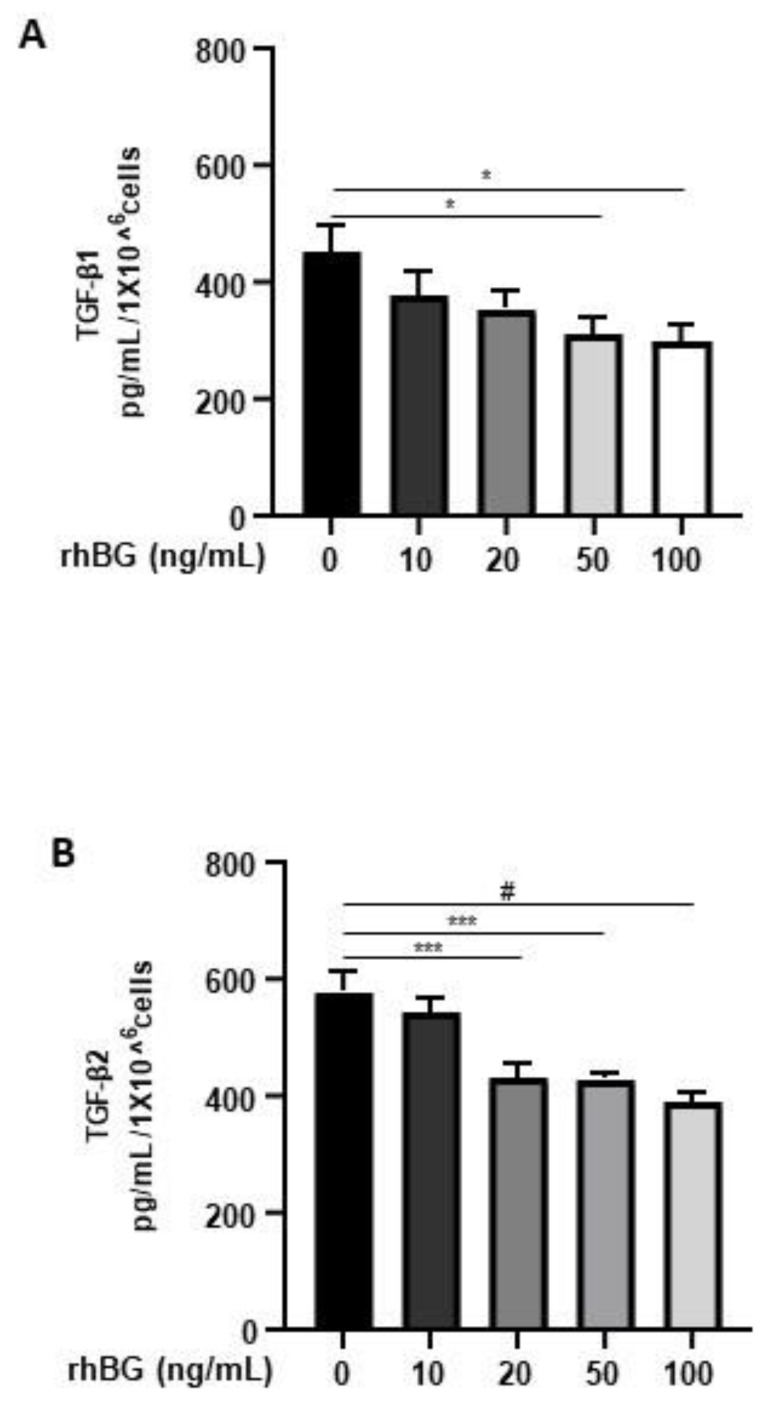
Dose-dependent effects of recombinant human BG on TGF-β1 and TGF-β2 secretion. Epithelial 12Z cells were treated with increasing concentrations of recombinant human betaglycan (rhBG (10–100 ng/mL)) for 48 h, and supernatants were analyzed by TGF-β1 (**A**) and TGF-β2 (**B**) ELISAs. rhBG treatment significantly decreased TGF-β1 and TGF-β2 levels in a concentration-dependent manner. Each bar represents the mean ± SEM of three independent experiments performed in duplicate. * *p* ≤ 0.05; *** *p* < 0.001; ^#^ *p* < 0.0001; rhBG, recombinant human betaglycan.

Membrane-bound BG is an important TGF-β co-receptor, whereas the soluble form functions as a TGF-β antagonist [16]. Thus, we next focused on the influence of BG on TGF-β1/-β2 secretion by 12Z cells. After treatment with soluble recombinant human BG (rhBG), we observed a moderate dose-dependent and significant reduction in the secretion of TGF-β1 (~20–33% with 20–100 ng/mL rhBG; Figure 4A) and TGF-β2 (~25–35% with 20–100 ng/mL rhBG; Figure 4B). This further confirms the role of BG in antagonizing TGF-βs.

### 3.3. The Impact of Matrix Metalloproteinases (MMPs) on BG Shedding

**Figure 5:**
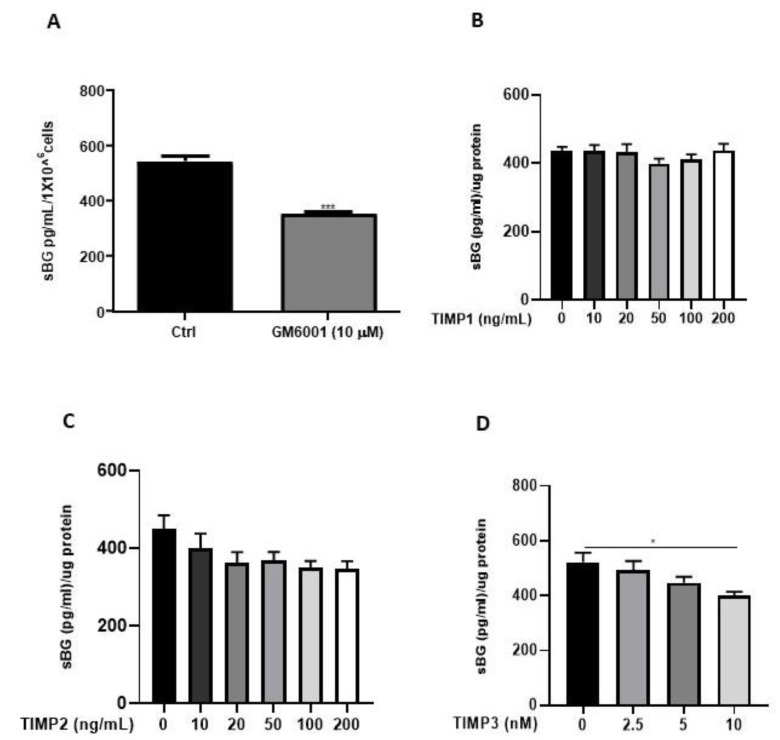
BG shedding is modulated by MMPs. Epithelial 12Z cells were treated with the broad-spectrum MMP inhibitor, GM6001 (10 µM) (**A**), or increasing concentrations of TIMP1 and TIMP2 (both with 10–200 ng/mL) (**B**,**C**) or TIMP3 (2.5–10 nM) (**D**) for 48 h, and supernatants were analyzed using sBG ELISA. Both GM6001 and TIMP3 treatment significantly attenuated BG shedding in 12Z cells whereas TIMP1/2 showed no significant effects. Each bar represents the mean ± SEM of three independent experiments performed in duplicate. * *p* ≤ 0.05, *** *p* < 0.001. sBG, soluble betaglycan.

We and others have previously reported that the membrane-type matrix metalloproteases (MT-MMPs) and tissue inhibitors of metalloproteinases (TIMPs) regulate BG shedding in certain cell types [27,41]. Accordingly, we investigated the influence of MMPs using the broad-range MMP inhibitor, GM6001, as well as TIMPs (Figure 5), on BG shedding. Epithelial 12Z cells treated with GM6001 led to a 35% reduction in BG shedding in 12Z cells (Figure 5A). Treatment with TIMP1 and TIMP2 had no substantial effects on BG shedding (Figure 5B,C). On the contrary, approximately 25% reduction in BG shedding was observed with 10 nM TIMP3, whereas lower doses had no significant effects (Figure 5D). Thus, inhibition of MMPs reduced BG shedding, confirming the involvement of MMPs.

**Figure 6:**
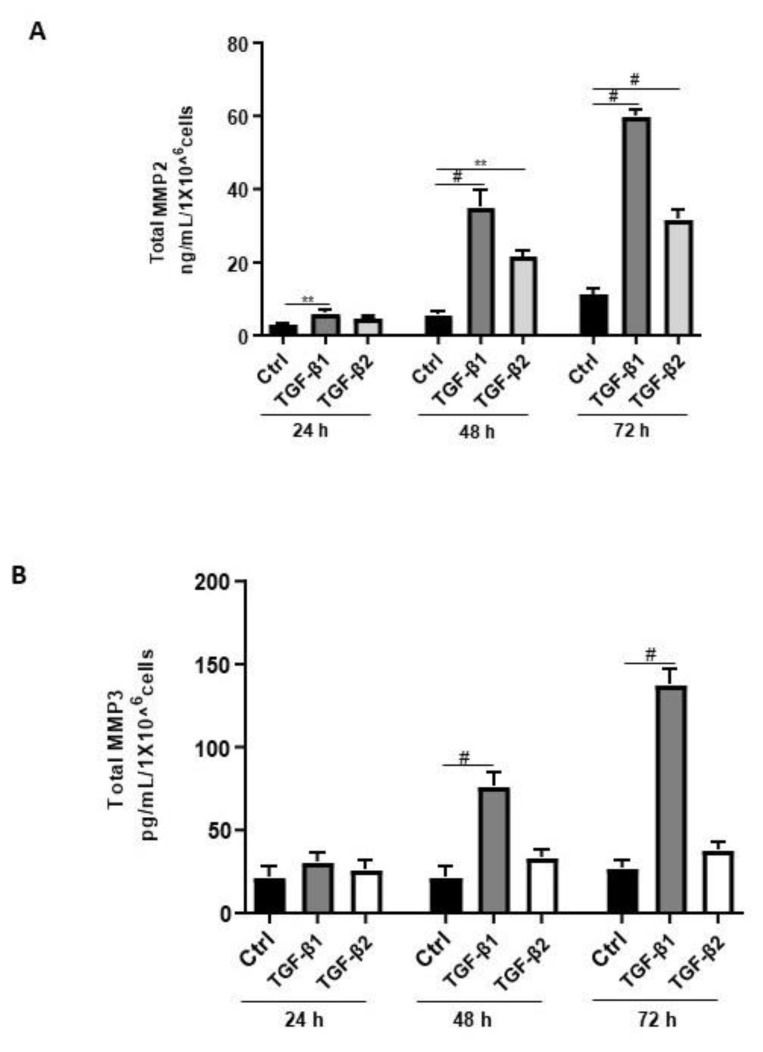
Time-dependent effects of TGF-β1 and TGF-β2 treatment on MMP2 and MMP3 secretion. Epithelial 12Z cells were stimulated with TGF-β1 or TGF-β2 (10 ng/mL) for 24, 48, and 72 h, and supernatants were analyzed by MMP2 (**A**) and MMP3 (**B**) ELISAs. Both TGF-β1 and -β2 stimulation of the epithelial cells significantly enhanced MMP2 secretion in a time-dependent manner. Additionally, TGF-β1 but not -β2 stimulation of the epithelial cells significantly increased MMP3 secretion in a time-dependent manner. Each bar represents the mean ± SEM of three independent experiments performed in duplicate. ** *p* < 0.01; ^#^ *p* < 0.0001; Ctrl, control.

Aberrant regulation of MMPs such as MMP2, MMP3, and MMP9 has been reported in endometriosis [42]. Thus, we sought to determine the effects of TGF-β1/β2 on the secretion of MMP2 and MMP3. Both TGF-β1 and -β2 induced MMP2 secretion in a time-dependent and significant manner (Figure 6A). In contrast, TGF-β1 but not TGF-β2 significantly and time-dependently induced MMP3 secretion in 12Z cells (Figure 6B). A comparison between the levels of secreted MMP2 and MMP3 revealed that 12Z cells secrete approximately 250 times more MMP2 than MMP3 (Figure 6A,B). Collectively, these results demonstrate that TGF-β1 but not TGF-β2 enhanced MMP2 and MMP3 secretion, consistent with its role in extracellular matrix remodeling and fibrosis [42].

### 3.4. Influence of TGF-β1/2 and BG on Wound Healing

**Figure 7:**
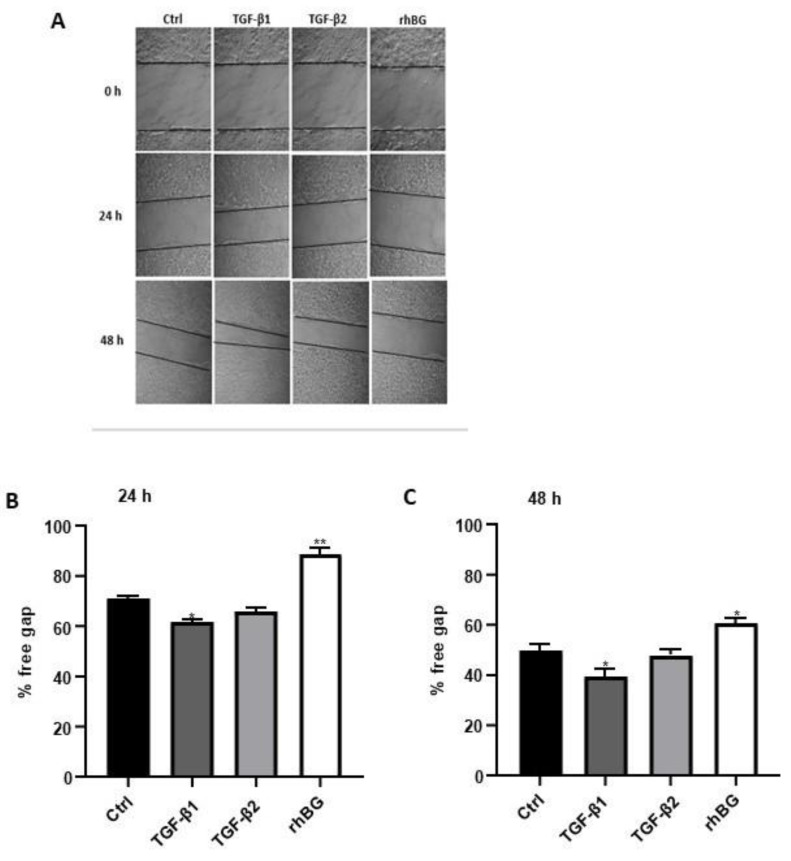
Effects of TGF-β1/β2 and BG on wound healing of 12Z cells were monitored by taking images at three different time points. Cells were cultured in 6-well plates and the confluent cell monolayer disrupted by scratching with a pipette tip. Cells were stimulated with or without 10 ng/mL TGF-β1, -β2 or 25 ng/mL rhBG and cell-free areas monitored at 0, 24, and 48 h by capturing images as described in methods (**A**). The results are presented as a percentage of the free gap following treatment with TGF-β1/β2 or rhBG using the ImageJ program analysis after 24 (**B**) and 48 (**C**) h. TGF-β1 but not TGF-β2 promoted migration of the endometriotic cells, whereas rhBG reduced migration of the cells. Each bar represents the mean ± SEM of three independent experiments performed in duplicate. * *p* ≤ 0.05; ** *p* < 0.01; rhBG, recombinant human betaglycan; Ctrl, control.

Context-dependent dysregulation of TGF-β signaling has been implicated in several wound healing pathologies [9,43]. Consequently, we investigated the influence of TGF-β1/2 and BG on wound healing of endometriotic 12Z cells (Figure 7). Compared to untreated cells, we found a moderate increase (~13% and 21% after 24 and 48 h, respectively) in wound closure after TGF-β1 treatment, whereas no significant effects were observed with TGF-β2 (Figure 7B,C). Conversely, approximately 25% reduction in the rate of wound closure was observed after BG treatment for 24 h and 48 h (Figure 7B,C).

### 3.5. Quantification of Serum and Endocervical Mucus sBG Levels in Patients with and without Endometriosis

Soluble BG levels were determined in sera from 238 patients with and without endometriosis (Table 2 and Table 3) and were similar at the different phases of the menstrual cycle (Table 3A), between women with and without endometriosis and with and without contraception (Table 3B).

In total, 182 endocervical mucus samples (n = 100 patients with and n = 82 without endometriosis) were analyzed for sBG (Table 2). Comparable to serum sBG, cycle independency was also found in endocervical mucus samples (Table 4A). Notably, the mean endocervical mucus sBG levels were significantly lower in endometriosis patients compared to controls, both in patients without contraception (~2-fold) as well as in patients using contraception (~3-fold; Table 4B). Moreover, further stratification based on menstrual phases demonstrated a similar reduction in sBG levels (~55%) in endometriosis compared to non-endometriosis cases during the secretory but not in the proliferative phase (Table 4C).

No significant associations between serum and endocervical mucus sBG levels and age, fertility or pain (dysmenorrhea, dyspareunia, dyschezia, and dysuria) were detected (Table 5C).

The utility of endocervical mucus sBG as a potential non-invasive diagnostic biomarker for endometriosis was calculated with a cut-off of 1052 pg/mL and demonstrated a sensitivity of 61% and a specificity of 63% (AUC = 0.66, 95% CI = 0.5797 to 0.7411, likelihood ratio = 1.667, *p* = 0.0002; Table S1; Supplementary material), suggesting that sBG is only a moderate sensitive/specific diagnostic marker for endometriosis.

## 4. Discussion

In the current study, we showed for the first time that TGF-β regulates the shedding of BG through the ALK-5–SMAD3 axis in human endometriotic cells and that the shedding process is modulated by MMPs. Furthermore, we found that rhBG treatment significantly reduced the secretion of TGF-βs as well as the rate of wound closure of endometriotic cells. Additionally, our findings indicated that TGF-β1 significantly enhanced the secretion of MMP2 and MMP3 and promoted the rate of wound closure of endometriotic cells. Remarkably, we also observed that sBG levels were significantly reduced in the endocervical mucus of patients with endometriosis compared to controls.

Betaglycan was originally identified as a non-essential ligand accessory receptor that bound TGF-β ligand and enhanced signaling, particularly of TGF-β2 [15,25]. More recent studies, however, have shown that BG binds multiple TGF-β superfamily members including inhibin A and exhibits important ligand-dependent and -independent roles, which extend far beyond its role as a simple TGF-β or inhibin A co-receptor [8,16,44].

### 4.1. Modulation of BG Shedding

Using Sertoli cells, we recently demonstrated that BG is crucial in TGF-β2 signaling [41]. In the present study, we further showed that both TGF-β1 and -β2 reduced shedding of BG in endometriotic epithelial cells with stronger effects of TGF-β2 compared to TGF-β1. This is possibly due to the observation that TGF-β2 requires recruitment and presentation to TBRII via BG since TGF-β2 has only a weak affinity for TBRII in the absence of BG [15,45].

We demonstrated for the first time that the canonical TGF-β pathway involving TGF-β/ALK-5/SMAD3 signaling is required in TGF-β-mediated reduction in the shedding of BG in endometriotic cells. Inactivation of SMAD3 but not of SMAD2 in endometriotic cells followed by treatment with TGF-β1/2 increased BG shedding, suggesting non-redundant SMAD3-dependent regulation of BG shedding. The distinct phenotypes of SMAD2/3-deficient mice [46,47] as well as the differential regulation of transcription of TGF-β1 target genes, for instance in SMAD2/3-deficient mice fibroblast cells [48], are indicative of non-compensatory functions, although redundancy in SMAD2/3 roles was also previously demonstrated in mice T cells [49]. Unlike membrane-bound BG that functions as an accessory protein and promotes binding of TGF-β to TBRII to enhance TGF-β signaling, sBG acts as a decoy for TGF-β ligands and antagonizes TGF-β signaling [25,50]. In our study, we showed that stimulation of endometriotic epithelial cells with recombinant BG resulted in a significant decrease of basal TGF-β1 and TGF-β2 levels. Although we observed a similar magnitude in the effects of BG on TGF-β1 and TGF-β2, Vilchis-Landeros et al. [45] reported a 10-fold higher anti-TGF-β potency of recombinant sBG against TGF-β2 compared to TGF-β1 in COS-1 and Mv1Lu cell lines. Previous studies showed that sBG binds and sequesters TGF-β1/2/3 and BMP2/4/7, suppressing signaling [15,45,50].

### 4.2. Involvement of MMPs in BG Shedding

MMPs are believed to play essential roles in the regulation of BG shedding in different cell types [27,41]. Thus, in this study we examined the effect of MMPs on BG shedding in endometriotic cells. Our hypothesis that MMPs modulate shedding of BG in endometrial cells is supported by findings from our inhibition studies because GM6001, a pan-MMP agonist, and TIMP3 significantly inhibited BG shedding in endometriotic cells. Although the mechanism of BG cleavage is still elusive, a study by Velasco-Loyden et al. [27] suggested that BG shedding is regulated by pervanadate, a tyrosine phosphatase inhibitor, and is partly mediated by MT1-MMP and/or MT3-MMP. TNF Protease Inhibitor 2 (TAPI-2), a tumor necrosis factor-converting enzyme (TACE) and MMP inhibitor, was found to suppress BG shedding [27,51], indicating the involvement of MMPs in the shedding process, which is consistent with our previous [41] and present findings. Markedly, the transmembrane-cytoplasmic BG fragment that remains after ectodomain shedding is stable and has important implications in TGF-β2 signaling [51]. TGF-βs are involved in the migration and invasion of cells in physiological processes such as embryo implantation as well as in pathological processes such as endometriosis [4,11,24]. Additionally, both in vitro and in vivo studies indicated that TGF-βs regulate MMPs [24,52]. Previously, we demonstrated that TGF-β1 and -β2 promoted secretion of both MMP2 and MMP9 in endometrial and endometriotic cells [52]. In the present study, we further showed that TGF-β1 stimulated MMP3 secretion in endometriotic cells and that the increase in both MMP2 and MMP3 secretion was time-dependent. Both MMP2 and MMP3 have been implicated in the pathogenesis of endometriosis [42,53].

### 4.3. Effects of TGF-β1/TGF-β2 and BG on Wound Healing

Members of the TGF-β superfamily are involved in wound healing and restoration of the endometrium following menstruation [43,54]. In this study, TGF-β1 but not TGF-β2 promoted wound closure of endometriotic cells, which is in agreement with previous studies implicating TGF-β1 in cell migration and wound healing [9,11]. Additionally, in our current study, rhBG moderately reduced the rate of wound closure of endometriotic cells possibly via migration. Previous reports showed that BG reduces the migration and invasion of different types of cancer cells [18,19,44,55,56,57].

### 4.4. Evaluation of BG as a Biomarker for Endometriosis

Studies on the pathology of endometriosis revealed disrupted TGF-β expression and signaling that in turn facilitated implantation and maintenance of ectopic endometrium [4,9,24]. Having demonstrated the essential roles of BG in TGF-β function in an in vitro endometriotic model, we investigated the potential role of BG in the pathophysiology of endometriosis in vivo with serum and endocervical mucus samples from patients with and without endometriosis. Recently, we and others have found that several proteins in the endocervical mucus might be correlated with endometriosis [6,7]. However, no significant correlations between serum and endocervical mucus sBG levels and menstrual phases were detected, consistent with a previous study with breast cancer patients [20]. Although we did not detect differences in serum sBG levels of patients with endometriosis versus without endometriosis, the endocervical mucus sBG levels were significantly lower in patients with endometriosis compared to patients without endometriosis. To our knowledge, this report is the first study to evaluate serum and endocervical mucus sBG levels in endometriosis. We suggest that reduced sBG levels in endocervical mucus of endometriosis patients may indicate enhanced TGF-β function characterized by increased cell migration and invasion, angiogenesis, and reduced apoptosis since sBG is known to antagonize TGF-β signaling [19,58].

In the past, disruption in BG expression or function has been linked to multiple pathologies including cancers [17,18,19,20]. In particular, BG was identified as a tumor suppressor in many tissue types, and down-regulation or loss of BG expression at the mRNA or protein levels correlated with increased tumor progression and poor prognosis in many cancers [17,19,20,32,33]. Decrease or loss of BG expression occurs via several molecular mechanisms, which include loss of heterozygosity at the BG gene locus and epigenetic silencing [59,60,61]. This is frequently associated with a loss of sensitivity to anti-proliferative TGF-β and/or inhibin-mediated activity, reduced NF-kB-mediated control of inflammation and immune response, in addition to increased cell migration and invasion, angiogenesis, and tumor progression along with reduced apoptosis [17,18,19,28,58,62].

**Figure 8:**
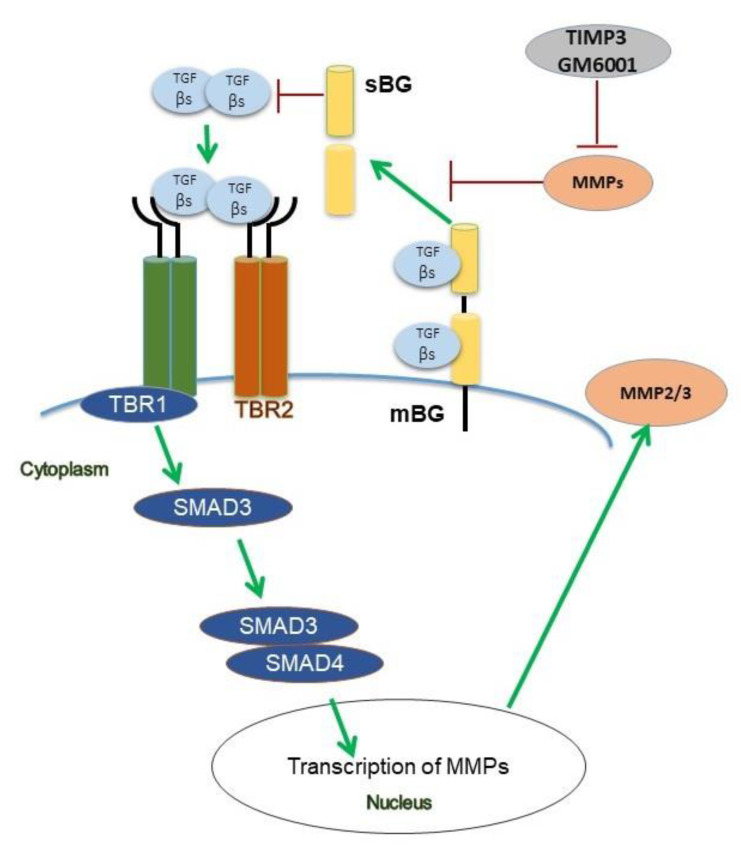
Scheme of TGF-β1 and TGF-β2 signaling under the influence of betaglycan (BG) and TIMP3 in human endometriotic epithelial cells. Binding of both ligands to the TGF-β receptor complex results in phosphorylation of Smad3 but not of Smad2. TGF-β1 and TGF-β2 increase secretion of MMP2, but only TGF-β1 increases the secretion of MMP3. TIMP3 and GM6001, a pan-MMP inhibitor, both reduce shedding of BG via inhibition of MMPs. In contrast to TGF-β1 which improves wound healing, soluble BG (sBG) exacerbates wound healing.

Collectively, both our patient data and functional in vitro studies highlight several novel aspects of BG in the context of TGF-β signaling (Figure 8) and endometriosis. Future studies will aim to recapitulate our present in vitro findings in an in vivo model and to determine the exact role of TGF-β-mediated effects on BG shedding in endometriosis.

## Figures and Tables

**Table 1:** List of primer sequences used for RT-qPCR.

Genes (Species)	Sequence (5′→3′)	Acc. No.	Size (bp)
SMAD2 (Hu)	ATCCTAACAGAACTTCCGCC fwd CTCAGCAAAAACTTCCCCAC rev	NM_001003652	481
SMAD3 (Hu)	GTCTGCAAGATCCCACCAGG fwd CTTGTCAAGCCACTGCAAAG rev	NM_001145102	237
GAPDH (Hu)	GACCCCTTCATTGACCTCAAC fwd GATGACCTTGCCCACAGCCTT rev	NM_001256799	561

Hu, human; Acc. No., accession number; fwd, forward; rev, reverse; bp, base pairs; GAPDH, Glyceraldehyde 3-phosphate dehydrogenase.

**Table 2:** Patient demographics and clinical characteristics.

	Serum Samples		Endocervical Mucus Sample	
	EM	*w/o* EM	EM	*w/o* EM
(n)	166	72	100	82
Mean age (SEM)	33.6 (0.6)	26.7 (0.9)	32.7 (0.7)	28.8 (0.8)
BMI (kg/m^2^)	23.3	22.9	23	22.7
Smoking (%)	24	19	28	23
Allergy (%)	59	64	51	48
Menstrual phase (n)				
Proliferative	28	19	27	19
Secretory	41	31	43	36
Menstruation	17	9	-	-
Unknown	80	13	30	27
Contraception use (n)				
Yes	68	43	30	31
No	98	29	70	51
Fertility (n)				
Yes	43	6	37	10
No	56	7	24	14
Unknown	67	59	39	58
Pain (n)				
Dysmenorrhea				
Yes	112	58	89	70
No	38	13	11	11
Unknown	16	1	-	1
Dyspareunia				
Yes	92	30	58	35
No	54	38	42	46
Unknown	20	4	-	1
Dyschezia				
Yes	60	8	45	29
No	95	60	55	53
Unknown	11	4	-	-
Dysuria				
Yes	33	7	29	15
No	125	63	71	66
Unknown	8	2	-	1
sBG detected (%)	166 (100)	72 (100)	96 (96)	81 (99)

EM, endometriosis; *w/o* EM, without endometriosis; SEM, standard error of the mean; BMI, body mass index; sBG, soluble betaglycan; Pain yes denotes mild to strong pain (scale 2–10); unknown means data not available.

**Table 3:** Soluble betaglycan levels in serum.

(A)	Proliferative	Secretory		Menstruation
n	47	72		26
Median age	28 ± 1.1	30 ± 0.9		30.5 ± 1.6
BMI (kg/m^2^)	22.8	24.1		22.2
Mean sBG (ng/mL)	61.8 ± 3.2	53.7 ± 2.8		52.7 ± 4.8
Range (ng/mL)	12–114	11–98		9–87
*_P_*	ns	ns		ns
(B)	Without contraception		With Contraception	
	EM ^a^	*w/o* EM ^b^	EM ^c^	*w/o* EM ^d^
n	98	29	68	43
Median age	35 ± 0.7	27 ± 1.5	30 ± 0.9	23 ± 1.0
BMI (kg/m^2^)	23.1	23.0	23.5	22.7
Mean sBG (ng/mL)	59.6 ± 2.2	60.3 ± 4.3	50.3 ± 3.1	48.7 ± 3.3
Range (ng/mL)	11–98	12–114	11–87	9–79
*_P_*	ns ^a,b^		ns ^c,d^	0.04 ^a,d^

n = 238; Data are the median ± SEM or mean ± SEM; SEM, standard error of the mean; sBG, soluble betaglycan; EM, endometriosis; *w/o* EM, without endometriosis; ns, not significant. 0.04 ^a,d^ means that comparison of EM ^a^ is significantly different from w/o EM ^d^.Analysis was performed using the Kruskal–Wallis test and the Mann–Whitney U-test.

**Table 4:** Soluble betaglycan levels in endocervical mucus.

(A)	Proliferative		Secretory	
n	46		79	
Median age	29 ± 1.0		30 ± 0.8	
BMI (kg/m^2^)	22.5		22.7	
Mean sBG (pg/mL)	2186 ± 407		1940 ± 267	
Range (pg/mL)	0–11,335		0–10,862	
*_P_*	ns		ns	
(B)	Without contraception		With Contraception	
	EM ^a^	w/o EM ^b^	EM ^c^	w/o EM ^d^
n	70	51	30	31
Median age	31 ± 0.8	29 ± 1.0	33 ± 1.3	23 ± 1.4
BMI (kg/m^2^)	22.4	23.2	23.6	22.1
Mean sBG (pg/mL)	1735 ± 243	3419 ± 454	912 ± 523	2573 ± 187
Range (pg/mL)	0–7865	0–11,335	0–4369	297–13,036
*_P_*	0.0103 ^a,b^		0.0007 ^c,d^	
(C)	With endometriosis		Without endometriosis	
	Proliferative ^a^	Secretory ^b^	Proliferative ^c^	Secretory ^d^
n	27	43	19	36
Median age	30 ± 1.3	34 ± 1.0	28 ± 1.5	28 ± 0.9
BMI (kg/m^2^)	22.7	22.5	22.2	23.2
Mean sBG (pg/mL)	1796 ± 418	1239 ± 260	2740 ± 782	2778 ± 463
Range (pg/mL)	0–7865	0–7473	260–11,335	0–10,862
*_P_*	ns ^a,b^		ns ^c,d^	0.0233 ^b,d^

n = 182; Data are the median ± SEM or mean ± SEM; SEM, standard error of the mean; sBG, soluble betaglycan; EM, endometriosis; w/o EM, without endometriosis; ns, not significant. 0.0103 ^a,b^ means that comparison of EM ^a^ is significantly different from w/o EM ^b^ (B). Analysis was performed using the Mann–Whitney U-test and the Kruskal–Wallis test.

**Table 5:** Correlations of cycle day, age, fertility, and pain with serum and endocervical mucus sBG levels.

	Serum sBG					
	n		Spearman r	Mean sBG (ng/mL)		*_P_*
		BMI (kg/m^2^)		Without Pain	With Pain	
Cycle day	145	23.1	−0.05			ns
Age	238	23.5	0.14			0.04
Fertility	112	24.0	0.07			ns
Dysmenorrhea	221	23.5		54.0	55.9	ns
Dyspareunia	214	23.4		56.3	53.6	ns
Dyschezia	223	23.5		56.5	50.2	ns
Dysuria	228	23.5		56.2	52.3	ns
	Endocervical mucus sBG					
	n		Spearman r	Mean sBG (pg/mL)		*_P_*
		BMI (kg/m^2^)		Without pain	With pain	
Cycle day	125	22.6	−0.10			ns
Age	182	22.9	−0.09			ns
Fertility	85	24.0	0.07			ns
Dysmenorrhea	181	22.9		2812	2110	ns
Dyspareunia	181	22.9		2128	1965	ns
Dyschezia	182	22.9		2438	1800	ns
Dysuria	181	22.9		2304	1906	ns

Cycle day (Days 1–32); without pain (pain scale = 0–3); with pain (pain scale 4–10); sBG, soluble betaglycan; ns, not significant. Analysis was performed using the Spearman’s r test and the Mann–Whitney U-test.

## Data Availability

We do not have publicly archived datasets.

## Supplementary Materials

The following supporting information can be downloaded at: https://www.mdpi.com/article/10.3390/biology11040513/s1, Table S1 Receiver operating chararcteristics (ROC) curve, Figure S1 Scheme of the experimental setup.
