# Supplementary material for: Role of Betaglycan in TGF-β Signaling and Wound Healing in Human Endometriotic Epithelial Cells and in Endometriosis
Source: Biology (Basel). 2022 Mar 26;11(4):513. doi: 10.3390/biology11040513 (PMC9027931; doi:10.3390/biology11040513)
Supplement: Supplementary file 1 [file biology-11-00513-s001.zip › biology-1623644-supplementary.pdf]

Table S1 Receiver operating characteristic (ROC) curve

	Area under the ROC curve				
	Area	0,6604			
	Std. Error	0,04118			
	95% confidence interval	0.5797 to 0.7412			
	P value	0,0002014			
	Control	82			
	Patient	100			
sBG (pg/mL)	Sensitivity	95% CI	Specificity	95% CI	Likelihood ratio
< 1024	0.59	0.4920 to 0.6813	0.6341	0.5261 to 0.7302	1.613
< 1036	0.6	0.5020 to 0.6906	0.6341	0.5261 to 0.7302	1.64
< 1052	0.61	0.5120 to 0.6998	0.6341	0.5261 to 0.7302	1.667
< 1113	0.62	0.5221 to 0.7090	0.6341	0.5261 to 0.7302	1.695
< 1168	0.62	0.5221 to 0.7090	0.622	0.5138 to 0.7192	1.64

1. Modulation of BG shedding and functions of BG

1.1. Effects of TGF-β1/-β2 on BG shedding (Fig.1) are mediated via

1.1.1. TGF-β receptor type I (ALK5, Fig.2) and transduced via

1.1.2. SMAD3 but not SMAD2 (Fig. 3)

1.2. Effects of recombinant BG on TGF-β1/-β2 (Fig.4)

1.3. Involvement of MMPs in BG shedding (Fig.5) blocked only by TIMP3 Fig.5D)

1.4. Effects of TGF-β1/-β2 on secretion of MMP2 and MMP3 (Fig. 6)

1.5. Effects of recombinant BG and TGF-β1/-β2 on wound healing (Fig. 7)

2. Evaluation of BG as a biomarker for endometriosis (Fig. 8, Tables 3,4, and S2)

Figure S1: Scheme of the experimental setup
